# Combined Surgical Approach to Intracranial and Extracranial Hemangiopericytoma: Case Report and Literature Review

**DOI:** 10.7759/cureus.7447

**Published:** 2020-03-28

**Authors:** Eduardo Morales Valencia, Luis Alberto Tavares de la Paz, Gabriel Santos Vázquez, Aarón Emanuel Serrano Padilla, Erick Moreno Pizarro

**Affiliations:** 1 Surgery, Hospital Regional de Alta Especialidad del Bajío, Leon, MEX; 2 Surgical Oncology, Hospital Regional de Alta Especialidad del Bajío, Leon, MEX; 3 Medicine, University of Guanajuato, Leon, MEX

**Keywords:** hemangiopericytoma, surgery, vascular tumor

## Abstract

Hemangiopericytoma (HPC) is a rare vascular tumor that was first described in 1942 and whose classification and treatment continue to develop. The proper classification for HPC is still under discussion, being considered a solitary fibrous tumor (SFT), classified as an aggressive biological form. The World Health Organization (WHO) has considered it to be part of extrapleural solitary fibrous tumors, however, neuropathologists still consider it to be an HPC when it is found in the central nervous system. We present a case of a patient with HPC of complex localization in the infratemporal fossa and middle floor of the skull base, which confirmed the diagnosis of HPC after resection by the craniofacial approach. Hemangiopericytomas are tumors that can present along with distant metastasis in 23% of cases even after resection. Surgery is the therapeutic basis; however, the still-controversial pathological classification of these vascular tumors and their uncertain biological behavior are the main reasons the ideal treatment continues to be investigated.

## Introduction

The first description of HPC was made in 1942 by South and Muray, who described it as a soft tissue neoplasm from Zimmerman's pericytes surrounding capillaries and post-capillary venules, with a branched "deer horn" vascular pattern [1-2]. Currently, it is known that HPC is a solitary fibrous tumor of the central nervous system, which represents 1% of vascular tumors, more common after the fourth decade of life, more frequently located in the extremities, pelvis, retroperitoneum, head and neck, meninges, lungs, and pleura, although cases not limited to these areas have been reported. Most tumors of fibrous origin are benign. The treatment for HPC is focused on surgical resection, however, these are more difficult to manage because there is a 15%-20% risk of local recurrence [3-4]. The vascular characteristics of this neoplasm have led to the investigation of treatments focused on angiogenesis, anti-interferon-α, or the use of pazopanib (a tyrosine kinase receptor inhibitor), having results of higher survival in small control groups [1].

The World Health Organization (WHO) in 2016 has created the combined term solitary fibrous tumor/hemangiopericytoma to describe such lesions. This term will probably be shortened in the upcoming WHO classification of central nervous system (CNS) tumors. Currently, in the WHO 2016 classification, it is classified into three grades, however, further studies are required to adjust this classification system, with the solitary fibrous tumor being one of the most important differential diagnoses [5].

## Case presentation

A 17-year-old female patient, with no significant history, described as having a right frontoparietal headache, which fades away on non-steroidal anti-inflammatory drugs (NSAIDs) administration. An increase in the volume of the right temporal region was added, which progressively increased its size. While exercising, she reported a sensation of intracranial fluid flow, denying nausea or vomiting, without loss of consciousness, preserved movement of the upper and lower extremities, without sensory deficit, and without visual alterations.

**Figure 1:**
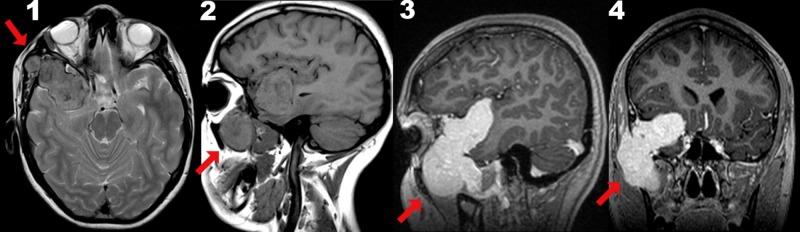
Magnetic resonance imaging Intracranial lesion from the Silvian Valley to the anterior temporal region, extension to the middle floor of the hourglass-shaped skull base, and the right extracranial temporal fossa Views. Left to right: 1 Transversal, 2 Sagital A, 3 Sagital B with gadolinium, 4 Coronal with gadolinium Red arrows 1-4: *An intracranial lesion can be observed from the Silvian Valley to the anterior temporal region, extension to the middle floor of the hourglass-shaped skull base, and right extracranial temporal fossa, with the remodeling of the posterior wall of the maxilla.* 3 and 4: *Intimate contact with lateral wall of the orbit in its intracranial portion, showing enhancement with gadolinium.*

Magnetic resonance imaging (MRI) was performed in November 2018, detecting an intracranial lesion from the Silvian Valley to the anterior temporal region, extending to the middle floor of the hourglass-shaped skull base, and right extracranial temporal fossa, remodeling of the posterior wall of the maxilla, intimate contact with the lateral wall of the orbit in its intracranial portion, showing enhancement with gadolinium. There was a normal neurological exploration in all areas (Figure 1).

**Figure 2:**
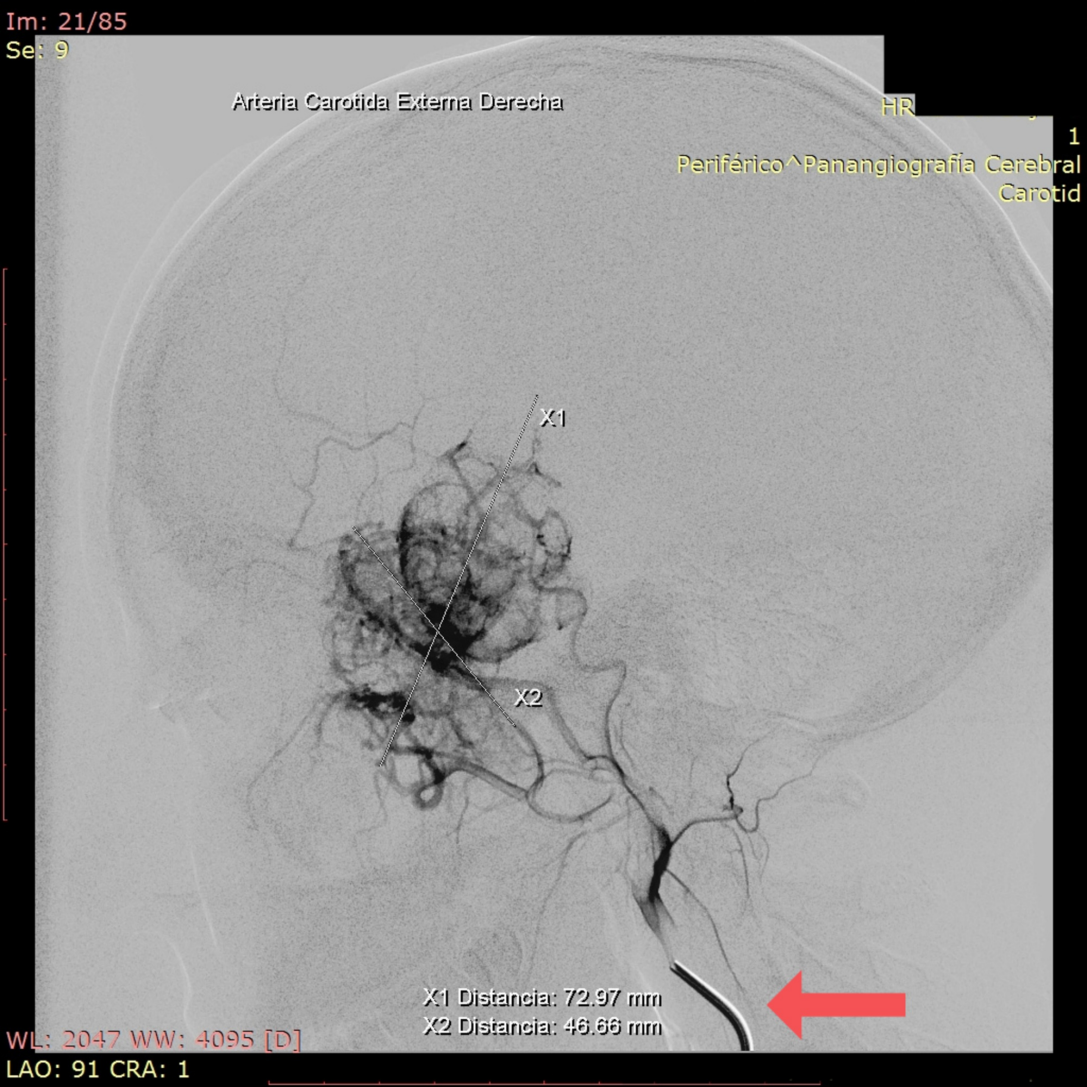
Cerebral angiography Right middle meningeal artery dependent tumor from the right internal maxillary artery Red arrow: *Right external carotid artery* Greater diameters of the tumor*: *X1: 72.97 mm, X2: 46.66 mm

**Figure 3:**
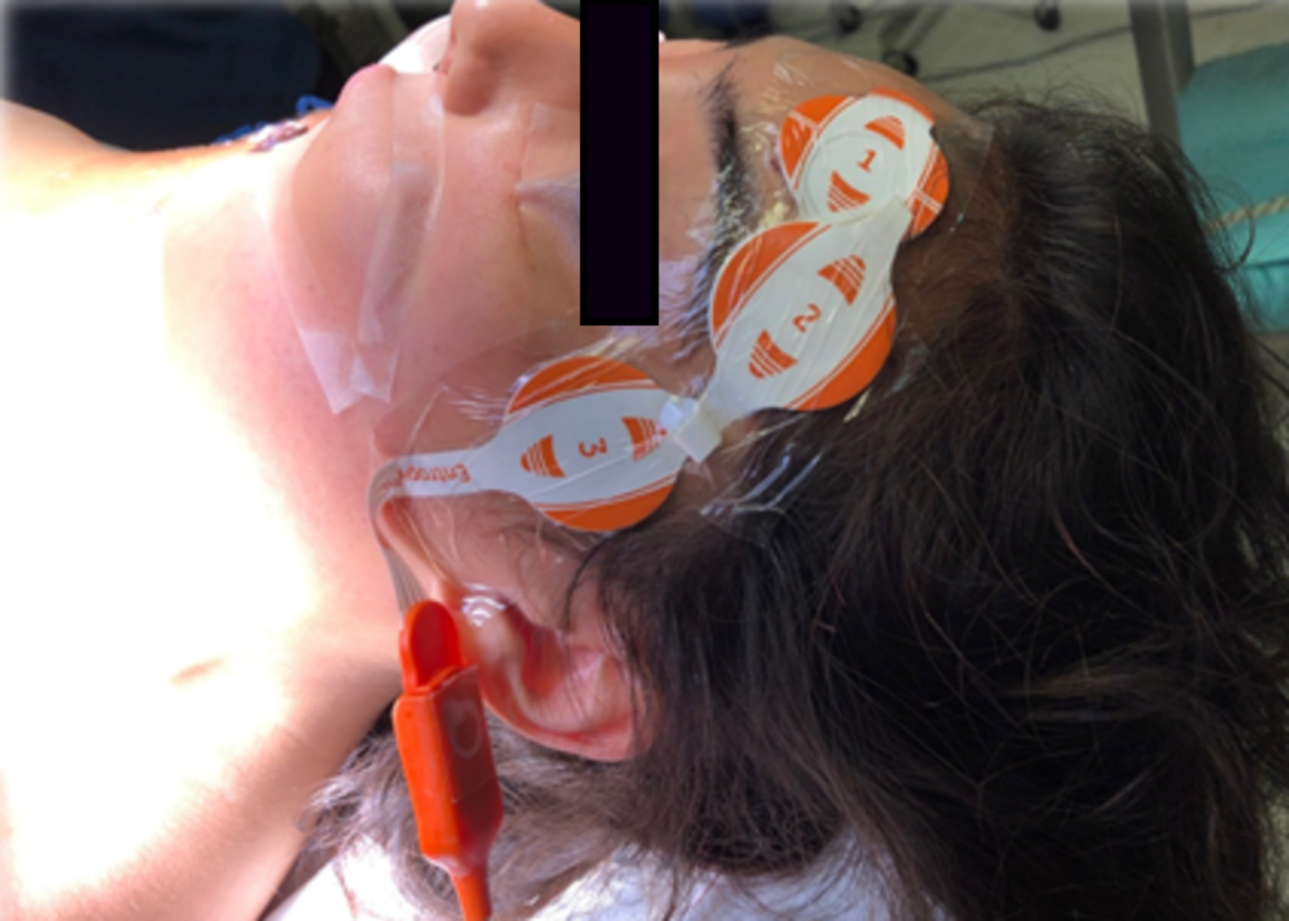
Entropy placement

**Figure 4:**
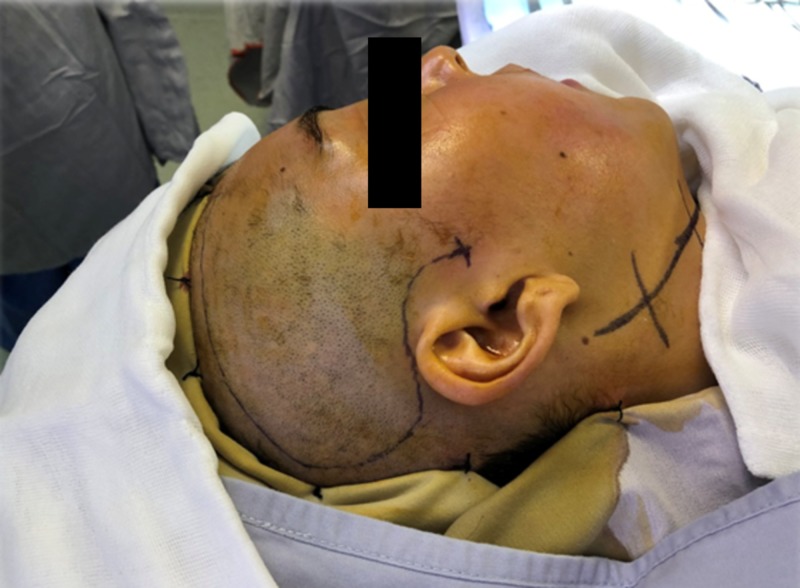
Surgical site marking

**Figure 5:**
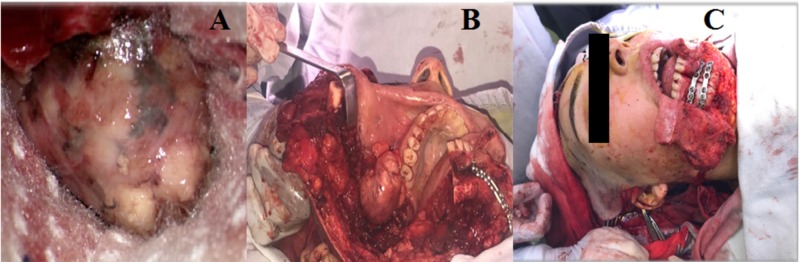
Images of the surgical procedure A: *Intracranial, extradural portion of the tumor; *B: *Extracranial tumor portion; *C: *Facial reconstruction with six-hole mini plates*

**Figure 6:**
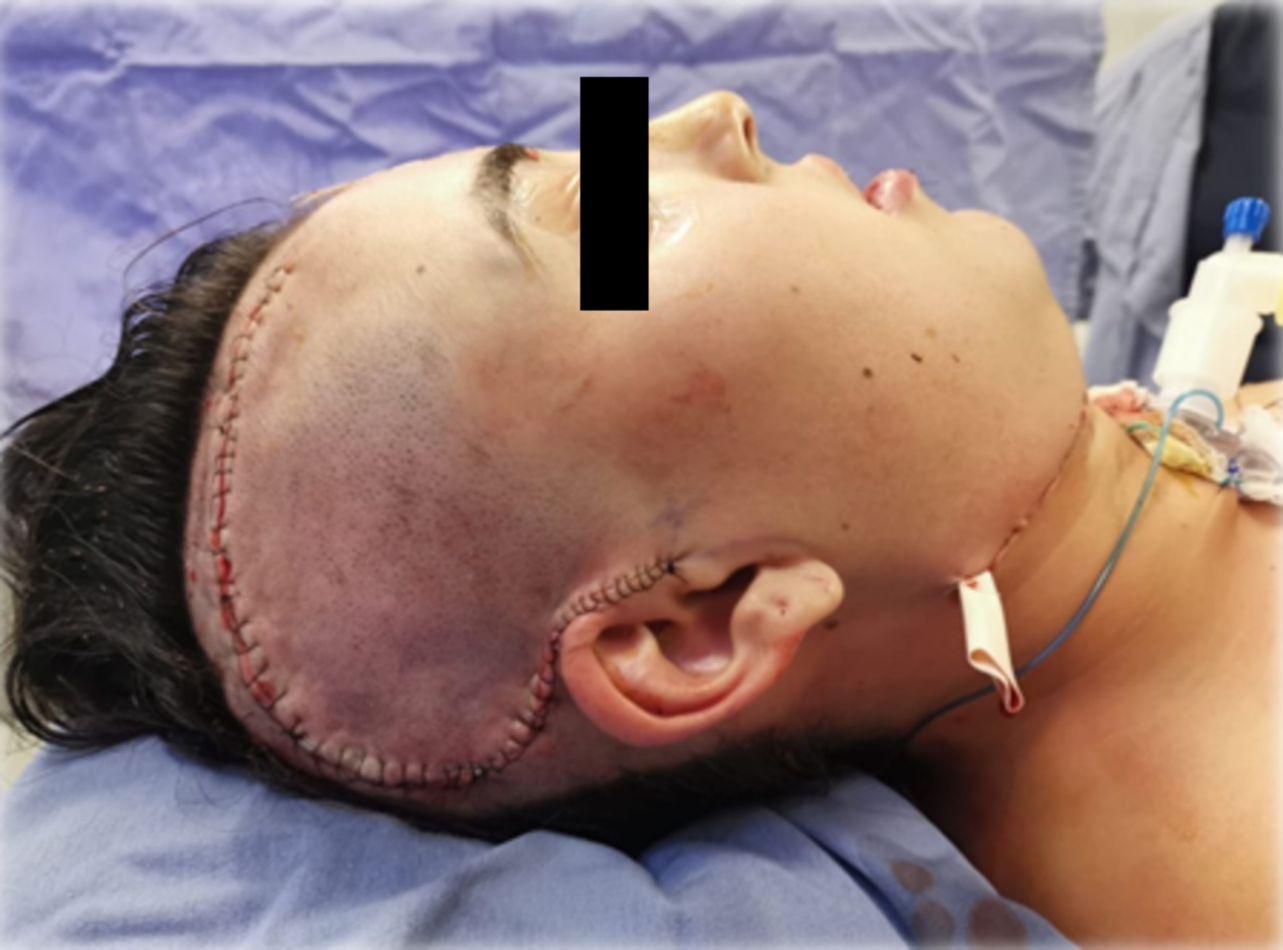
Final aesthetic result

A surgical plan was determined and in January 2019, a cerebral angiography was programmed. In July 2019, tumor embolization was requested (Figure 2), and surgery was performed on the following day, starting with a tracheotomy and pterional craniotomy, performing tumor resection in the intradural temporal region and resection by the transfacial approach (mandibular osteotomy, tumor resection, and mandibular osteosynthesis) (Figures 3-6).

**Figure 7:**
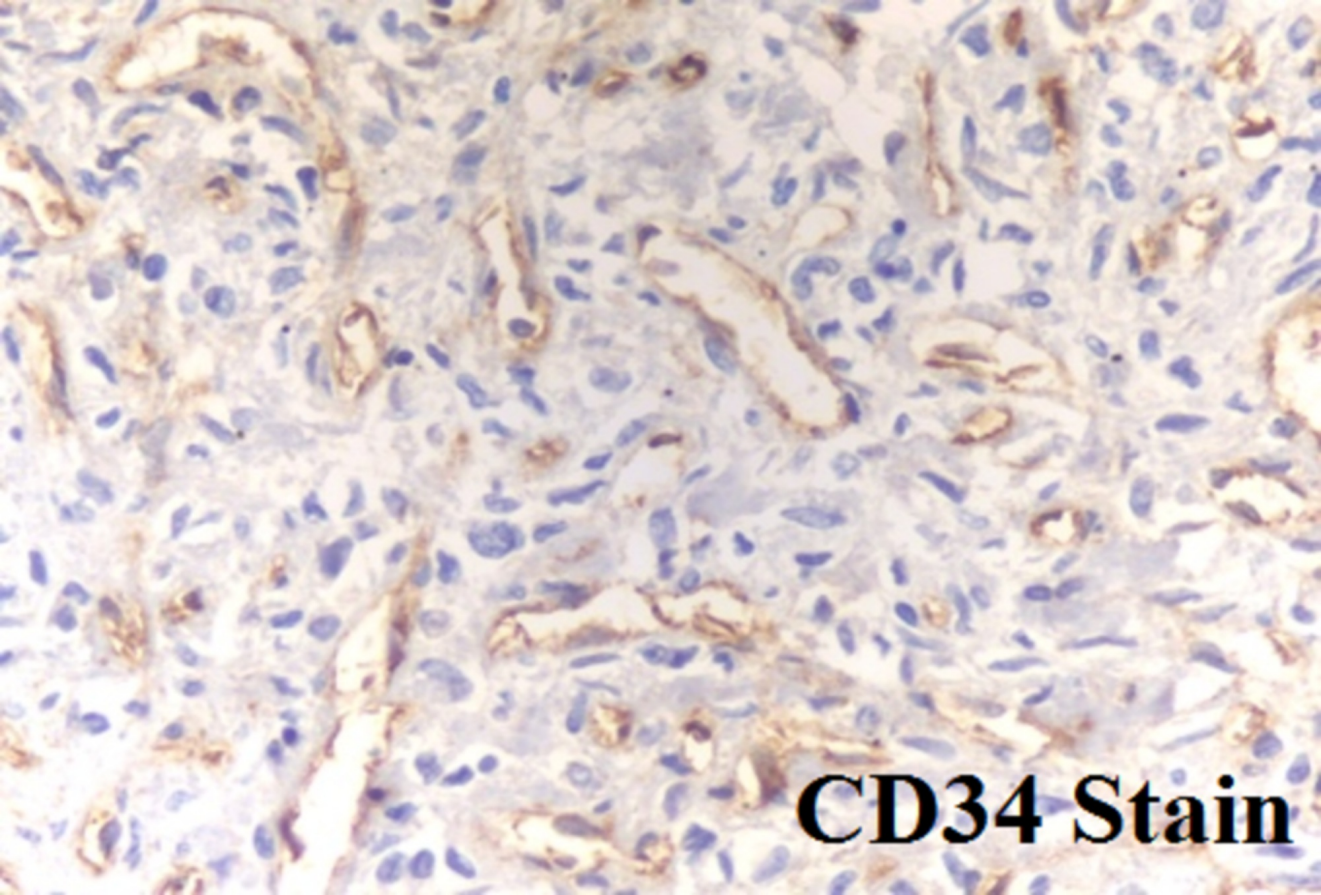
CD34-positive histopathology report

The histopathology report of the tumor describes a hemangiopericytoma, showing positivity for CD34 and vimentin, with negativity for CD31 and S-100 (Figure 7).

In the immediate postoperative period, she was admitted to the intensive care unit (ICU), which she attended favorably, without complications, with a five-day stay. A salivary gland fistula occurred, which was managed conservatively. From August 2019 to February 2020, she was undergoing radiotherapy treatment. She will continue her follow-up in oncology-surgery appointments.

## Discussion

HPCs are highly vascular tumors of mesenchymal, non-meningothelial lineage. They are very rare vascular tumors and comprise 1% of these.

The symptomatology of HPC is variable and depends on the location in which it occurs, with symptoms reported such as pain, paresthesia, abnormal deep tendon reflexes, nausea, vomiting, or altered gait, among others; in comparison with our clinical case, the patient only refers to a frontoparietal headache [6-7].

The diagnosis of this entity is by exclusion. On imaging study, its distinction from a meningioma can be difficult due to similar characteristics without depending on the location. HPCs are distinguished from meningiomas by their hypercellularity, higher mitotic index, and microscopic tendency to bulge into unexploded vascular lumens through the endothelium [8]. Identification with the surgical piece is ideal, the biopsy is not recommended due to the risk of bleeding secondary to the high vascularity.

The immunohistochemical study supports the definitive diagnosis, showing positivity for CD34 and vimentin, with negativity for CD31 and S-100, all characteristics of HPC (Figure 6) [2,5].

The proper classification of HPC is still under discussion, being considered a solitary fibrous tumor (SFT), cataloged as an aggressive biological form. The WHO has considered it as part of extrapleural solitary fibrous tumors, however, neuropathologists still consider it with the term hemangiopericytoma when it is found in the central nervous system, which is why it is necessary to compare both entities as seen in Table 1 [9]. The treatment of choice is total tumor resection and adjuvant radiation therapy is highly recommended, however, the rate of recurrence is high so new biologically focused therapies have been considered as part of the elective treatment [10].

**Table 1:** Comparison between hemangiopericytoma and solitary fibrous tumor

Tumor	Hemangiopericytoma	Solitary Fibrous Tumor
Date of description	1942	1870
Progenitor cell	Zimmerman pericytes	Intercapillary mesenchymal cell stromal, from submesothelial pericytes
Histological pattern	Vascular branches in deer antler form.	Uniform cells, with collagen, tapered, in interlaced fascicles. Pattern known as patternless pattern
Biological behavior	Benign 30%	Benign 80%
Anatomical region affected	Anywhere, predominantly lower extremities, retroperitoneum, head, and neck	Pleura dominates, can appear anywhere.
Immunohistochemical markers	Actin, tropomyosin, CD34	CD34, CD99, Bcl-2, Vimentin
Age and gender most frequently seen	20-70 years old 1:1 Female:Male	50-70 years old 1:1 Female:Male
Symptomatology	Local growth with compression symptoms	Chest pain, cough, dyspnea, local compression symptoms
Paraneoplastic syndromes	Non-associated	Hypertrophic osteoarthropathy Doege-Potter syndrome
Pathological description	Most often described by neuropathologists	Mostly accepted in general
Treatment of choice	Surgical resection	Surgical resection
5-year survival rate	Not described, most die within the first year	80-90%

The rate of extraneural metastasis is high, even higher than other primary tumors of the central nervous system, increasing the risk to 64% from 15 years after diagnosis [11].

## Conclusions

HPC and SFT are rare tumors, with no particular data for suspicion in the initial approach of a patient with an occupational injury. HPC in the central nervous system and SFT at the pleural level are considered more frequent. Surgical treatment is the only option with better survival. It is important to follow up and describe cases like this because of their low frequency and the little information about them in the global literature.
